# Hitting a HOMER: Epidemiology to the Bedside when Evaluating for Stereotactic Ablative Radiotherapy

**DOI:** 10.1164/rccm.201910-1933ED

**Published:** 2020-01-15

**Authors:** David M. DiBardino, Neal Navani

**Affiliations:** ^1^ Division of Pulmonary, Allergy and Critical Care Medicine University of Pennsylvania Philadelphia, Pennsylvania; ^2^ University College London Respiratory and; ^3^ Department of Thoracic Medicine University College London Hospital London, England

In this issue of the *Journal*, Martinez-Zayas and colleagues (pp. 212–223) report and validate a novel prediction model (HOMER) to calculate the probability of patients with non-small cell lung cancer (NSCLC) having mediastinal lymph node involvement (1). Determining a patient’s likelihood of lymph node metastasis is paramount in determining the stage of lung cancer and therefore appropriate treatment options. Clinical staging, including imaging modalities and biopsy techniques, remains a challenge and frequently falls short of surgical staging, depending on how aggressive the preoperative evaluation is (2). Accurate staging has been associated with improved survival and remains a huge emphasis in the care of patients with lung cancer (3). The study by Martinez-Zayas and colleagues is the first to derive and validate a risk model aimed at discriminating between the most clinically useful forms of nodal disease in patients who were both surgical and nonsurgical candidates: N0, N1, and N2/3 disease.

The authors should be commended for the statistical rigor used to derive and validate their model. Covariates used to develop the model were pragmatic, clinically relevant, and appropriately limited by the last common outcome. By externally validating their prediction model at other medical centers, the authors offer a model with the possibility of geographic stability for patients with NSCLC without T4 tumors or distant metastasis, after adjusting for the local institution’s population. The authors further supported their model with temporal validation to show stability over time (4). HOMER therefore has the potential to be generalizable in both the short term and the long term for patients with NSCLC seeking treatment at well-practiced thoracic oncology centers that use systematic endobronchial ultrasound-guided transbronchial needle aspiration (EBUS TBNA) lymph node staging. To carry out the systematic EBUS lymph node staging that the output of HOMER applies to, an examination of the intrathoracic nodes is required by EBUS, beginning with contralateral N3 nodes, followed by N2 and then N1 lymph nodes. Any lymph node measuring ≥5 mm in short axis is sampled, aiming for a minimum of three N2/3 lymph node stations sampled per procedure (5).

There are several clinically useful applications of HOMER. Assuming a patient is not a surgical candidate, the preferred treatment for N0 disease is definitive stereotactic ablative radiotherapy (SABR) and will not be confirmed surgically. Therefore, making an accurate clinical prediction of this disease state is crucial (6). Previous work was not able to discriminate between patients with N0 and N1 disease (7). HOMER presents two exciting ways to bring evidence-based decision making to these patients. First, the model can help predict the pretest probability that an EBUS TBNA will detect NSCLC, based on widely available clinical and radiographic data. As pointed out elegantly in the discussion, this can allow a more objective discussion about the risk and benefit of requiring an EBUS TBNA before SABR. As the low risk of complication is approached by the predicted probability of lymph node metastasis detected by EBUS TBNA, one can more confidently consider avoiding invasive mediastinal staging. This is especially relevant for patients at increased risk for complications during bronchoscopy. This may also be useful for patients with confirmed NSCLC from a transthoracic needle biopsy with radiographic N2/N3 disease who are at extremely high risk for bronchoscopy.

The other way HOMER can be used for these patients is to calculate a posttest probability of N1 disease in a patient being considered for SABR who has a negative EBUS TBNA. Current guidelines appropriately lean toward cytologic or pathologic confirmation for mediastinal staging. They suggest preoperative invasive mediastinal staging in patients with NSCLC unless the tumor is T1 (<3 cm) and peripheral, and the mediastinal lymph nodes are radiographically negative by computed tomography and positron emission tomography (7). This recommendation is based on a low false-negative rate (i.e., lymph node metastasis) in this patient population, as determined by older descriptive studies (8). Importantly, there are more recent data to support occult lymph node metastasis and a limited sensitivity for EBUS TBNA in similar patient populations (9). After making an assumption about the sensitivity of EBUS TBNA, a clinician can calculate a posttest probability, using HOMER, as is also demonstrated in the discussion. The ability to have an objective probability of N1 disease after a negative EBUS TBNA can assist the multidisciplinary lung cancer team when weighing the harm of SABR with occult N1 disease versus the harm of a larger radiation field and the addition of chemotherapy for presumed N1 disease.

As the authors warn in the discussion, HOMER should not be used to calculate the sensitivity of EBUS TBNA or the pretest probability of nodal disease, as there was no gold standard (i.e., surgical lymph node dissection) to compare with EBUS TBNA. Therefore, one must often put the model in the context of an assumed EBUS TBNA sensitivity, which is probably dependent on technique, lymph node size, necrosis, and tumor cellularity of each nodal metastasis. As mentioned here, EBUS TBNA may not be highly sensitive for NSCLC in radiographically normal lymph nodes (8). Clinicians may need to integrate HOMER with other observational studies associating standardized uptake value (SUV_max_) of the primary tumor, adenocarcinoma histology, non-lower lobe tumors, and tumor size with occult lymph node metastasis after negative preoperative positron emission tomography/computed tomography (10–13).

HOMER affords the opportunity to integrate data-driven decision making into our NSCLC staging and treatment decisions, much in the way we use probability to guide the management of lung nodules (14). The model’s performance could even be further refined as more data become available for patients with N1 disease. There are exciting ways to imagine an extended data set and similar methods being employed to predict other clinically meaningful outcomes in NSCLC. For nonsurgical patients, can we model the probability of long-term clinical response, using SABR, after a negative systematic EBUS? For surgical patients, can a model to predict occult lymph node metastasis after a negative systematic EBUS be similarly derived and validated? HOMER is an excellent example of using evidence collected from current practice to rigorously create a novel prediction tool to aid future clinical decisions. It is an important guide in practice and in principle, as we continue to strive for more evidence-based and data-driven care for patients with lung cancer.

## Supplementary Material

Supplements

Author disclosures
