# Supplementary material for: Hitting a HOMER: Epidemiology to the Bedside when Evaluating for Stereotactic Ablative Radiotherapy
Source: Am J Respir Crit Care Med. 2020 Jan 15;201(2):136–8. doi: 10.1164/rccm.201910-1933ED (PMC6961734; doi:10.1164/rccm.201910-1933ED)
Supplement: Supplements [file rccm.201910-1933ED.html]

Hitting a HOMER: Epidemiology to the Bedside when Evaluating for Stereotactic Ablative Radiotherapy | American Journal of Respiratory and Critical Care Medicine

- disclosures.pdf (894 KB)
